# Editorial: Emerging mechanisms in neuroinflammation: potential therapeutic targets for neurodegenerative diseases

**DOI:** 10.3389/fncel.2026.1908534

**Published:** 2026-07-10

**Authors:** Junhui Wang, Hongxing Wang, Dirk M. Hermann

**Affiliations:** 1 Lunenfeld-Tanenbaum Research Institute, Mount Sinai Hospital, Toronto, ON, Canada; 2 Department of Neurology, Xuanwu Hospital, Capital Medical University, Beijing, China; 3 Department of Neurology, University Hospital Essen, University of Duisburg-Essen, Essen, Germany

**Keywords:** cytokines, mitochondria, neurodegenerative, neuroinflammation, neuroprotection

Neurodegenerative diseases (NDs), including Alzheimer's disease (AD), Parkinson's disease (PD), Huntington's disease (HD), and amyotrophic lateral sclerosis (ALS), represent a major and growing global health challenge. These disorders are characterized by the progressive loss of neuronal structure and function within specific regions of the central nervous system, ultimately leading to cognitive decline, motor impairment, behavioral abnormalities, and reduced quality of life (Lamptey et al., 2022). Despite extensive research efforts, effective disease-modifying therapies remain limited, highlighting the urgent need for a deeper understanding of the underlying pathogenic mechanisms.

The etiology of neurodegenerative diseases is complex and multifactorial. A wide range of cellular and molecular processes have been implicated in disease initiation and progression, including abnormal protein aggregation, mitochondrial dysfunction, oxidative stress, excitotoxicity, disrupted synaptic communication, impaired proteostasis, cellular senescence, and dysregulated ion homeostasis (Yang, 2025; Wilson et al., 2023). Increasing evidence suggests that these pathological events do not occur in isolation but rather interact within a dynamic network that ultimately drives neuronal dysfunction and degeneration.

Among these interconnected mechanisms, neuroinflammation has emerged as a critical contributor to the pathogenesis of neurodegenerative diseases. Once considered merely a secondary response to neuronal injury, neuroinflammation is now recognized as an active and often early participant in disease progression. Both clinical observations and experimental studies have demonstrated that inflammatory processes are closely associated with neurodegeneration across multiple disease contexts (Zhang et al., 2023). Activation of resident immune cells, including microglia and astrocytes, together with the recruitment of peripheral immune components, can profoundly influence neuronal survival and function (Latour and McGavern, 2026).

Neuroinflammatory responses are triggered and sustained by a diverse array of signaling molecules, including damage-associated molecular patterns (DAMPs), cytokines, chemokines, reactive oxygen species, and other inflammatory mediators

(García-Domínguez, 2025). While acute inflammatory responses may initially serve protective functions by promoting tissue repair and the clearance of cellular debris, chronic or dysregulated neuroinflammation can become detrimental, amplifying neuronal damage and accelerating disease progression. Advances in transcriptomic, proteomic, and single-cell technologies have further revealed remarkable heterogeneity among glial and immune cell populations, highlighting the complexity of inflammatory signaling networks within the diseased nervous system.

The cyclic GMP–AMP synthase (cGAS) is a cytosolic pattern recognition sensor that detects cytosolic DNA derived from pathogens or damaged host cells. Upon recognizing double-stranded DNA, cGAS generates the second messenger cGAMP, which activates downstream inflammatory signaling pathways, including type I interferon responses. By sensing both exogenous and endogenous DNA, such as mitochondrial and nuclear DNA released during cellular stress, the cGAS pathway serves as a critical mediator of innate immunity and neuroinflammation (Yu and Liu, 2021). A recent study has suggested the role of global cGAS–STING signaling in various neurological and neuroinflammatory diseases and the potential contribution of cGAS–STING to neurodegenerative diseases via brain endothelial cells (Sun et al., 2024).

Over the past decade, substantial progress has been made in elucidating the molecular and cellular pathways that govern neuroinflammatory processes. These discoveries have uncovered numerous potential therapeutic targets, including inflammatory signaling cascades, immune cell activation states, metabolic pathways, and mechanisms of intercellular communication. Nevertheless, many fundamental questions remain regarding the precise roles of neuroinflammation during different stages of disease progression and the extent to which specific inflammatory pathways may be exploited for therapeutic benefit.

This Research Topic, *Emerging mechanisms in neuroinflammation: potential therapeutic targets for neurodegenerative diseases*, aims to provide a comprehensive overview of recent advances in our understanding of neuroinflammatory mechanisms and their implications for neurodegenerative disorders. By bringing together contributions from diverse fields, including neuroscience, immunology, molecular biology, and translational medicine, this Research Topic highlights novel insights into the complex interactions between the nervous and immune systems and explores emerging strategies for therapeutic intervention. Nine publications are included in the Research Topic with five review articles and four original research articles.

In their study, Paldino et al. investigated the role of the CD47–SIRPα signaling pathway in HD, a pathway known to regulate immune responses and phagocytosis. The authors found that CD47 expression was reduced in striatal neurons of HD mice, whereas SIRPα expression was increased. Disruption of CD47–SIRPα signaling was associated with decreased activation of the phosphatase SHP-1 and increased neuronal STAT1 expression, suggesting enhanced inflammatory signaling. These findings identify impaired CD47–SIRPα-mediated regulation of neuroinflammation as a potential contributor to neuronal damage in HD and highlight this pathway as a possible therapeutic target for neuroprotection.

In a large cohort of patients with multiple system atrophy (MSA), Chen, Chen et al. examined the relationship between circulating inflammatory cytokines and clinical manifestations. They found that serum TNF-α levels were significantly elevated in MSA patients compared with healthy controls and showed a moderate diagnostic value for distinguishing MSA from controls. Both TNF-α and IL-6 were associated with anxiety symptoms, while IL-6 was additionally linked to disease severity and functional impairment. These findings support a role for peripheral inflammation in MSA pathogenesis and suggest that TNF-α may serve as a potential biomarker for disease identification.

Another study led by Park et al. investigated the therapeutic potential of near-infrared photobiomodulation (PBM) in a scopolamine-induced model of Alzheimer's disease-like cognitive impairment. PBM improved learning and memory performance, reduced neuronal cell death in the hippocampus, and modulated MAPK signaling and astrocyte activation. These findings suggest that PBM may exert neuroprotective effects through the regulation of neuroinflammatory pathways and astrocyte function, highlighting a promising non-invasive therapeutic approach for neurodegenerative disorders.

A study established a novel tri-culture model comprising vascular endothelial cells, neurons, and microglia to investigate the mechanisms underlying hypertension-related depression (HD) (Zhao et al.). The model successfully recapitulated key pathological features observed in HD, including endothelial dysfunction, neuroinflammation, neuronal injury, microglial activation, and disturbances in monoamine neurotransmitters. Mechanistically, these changes were associated with activation of the TLR4/NF-κB inflammatory pathway. By mimicking the interactions between vascular and neural components, this *in vitro* model provides a valuable platform for studying neuroinflammatory processes and evaluating potential therapeutic strategies for HD.

In addition to the research articles, we collected four review articles as well. Research on neuroinflammation in Parkinson's disease has expanded rapidly, identifying microglial activation, oxidative stress, α-synuclein pathology, and gut–brain interactions as key drivers of disease progression (Chen, Xie et al.). Mitochondrial dysfunction, lysosomal impairment, and neuroinflammation form an interconnected pathological network that contributes to early Parkinson's disease pathogenesis and represents a promising target for early diagnosis and intervention (Lv et al.). Distinct natural killer cell subsets play important immunoregulatory roles in multiple sclerosis by modulating innate and adaptive immune responses involved in disease development (Aghaee et al.). Insulin-like growth factor-2 (IGF2) has emerged as a potential therapeutic target for Alzheimer's disease due to its critical role in synaptic plasticity, memory formation, and neuroprotection (Chen, Lu et al.).

Collectively, these studies underscore the growing recognition that targeting neuroinflammation may offer promising opportunities to slow disease progression and improve outcomes for patients affected by neurodegenerative diseases. Increasing evidence suggests that neuroinflammatory processes play a central role in the onset and progression of disorders such as Alzheimer's disease, Parkinson's disease, and amyotrophic lateral sclerosis. By modulating inflammatory pathways, reducing chronic immune activation, and promoting a more supportive neural environment, emerging therapeutic strategies may help preserve neuronal function, delay neurodegeneration, and enhance patients' quality of life. As research continues to elucidate the complex interactions between the nervous and immune systems, interventions aimed at neuroinflammation are becoming an increasingly important focus in the development of disease-modifying treatments.
